# Evaluation of STAT3 Signaling in ALDH+ and ALDH+/CD44+/CD24− Subpopulations of Breast Cancer Cells

**DOI:** 10.1371/journal.pone.0082821

**Published:** 2013-12-23

**Authors:** Li Lin, Brian Hutzen, Hsiu-Fang Lee, Zhengang Peng, Wenlong Wang, Chongqiang Zhao, Huey-Jen Lin, Duxin Sun, Pui-Kai Li, Chenglong Li, Hasan Korkaya, Max S. Wicha, Jiayuh Lin

**Affiliations:** 1 Divison of Cardiology, Department of Internal Medicine, Tongji Hospital, Tongji Medical College, Huazhong University of Science and Technology, Wuhan, Hubei, People's Republic of China; 2 Center for Childhood Cancer, The Research Institute at Nationwide Children’s Hospital, Department of Pediatrics, Columbus, Ohio, United States of America; 3 Molecular, Cellular, and Developmental Biology Program, The Ohio State University, Columbus, Ohio, United States of America; 4 Department of Pharmaceutical Sciences, College of Pharmacy, The University of Michigan, Ann Arbor, Michigan, United States of America; 5 Medical Technology Division, School of Allied Medical Professions, Columbus, Ohio, United States of America; 6 Department of Medical Laboratory Sciences, College of Health Sciences, University of Delaware, Newark, Delaware, United States of America; 7 Division of Medicinal Chemistry and Pharmacognosy, College of Pharmacy, The Ohio State University, Columbus, Ohio, United States of America; 8 Department of Internal Medicine, The University of Michigan Comprehensive Cancer Center, Ann Arbor, Michigan, United States of America; Medical Center at Seattle, United States of America

## Abstract

**Background:**

STAT3 activation is frequently detected in breast cancer and this pathway has emerged as an attractive molecular target for cancer treatment. Recent experimental evidence suggests ALDH-positive (ALDH^+^), or cell surface molecule CD44-positive (CD44^+^) but CD24-negative (CD24^−^) breast cancer cells have cancer stem cell properties. However, the role of STAT3 signaling in ALDH^+^ and ALDH^+^/CD44^+^/CD24^−^ subpopulations of breast cancer cells is unknown.

**Methods and Results:**

We examined STAT3 activation in ALDH^+^ and ALDH^+^/CD44^+^/CD24^−^ subpopulations of breast cancer cells by sorting with flow cytometer. We observed ALDH-positive (ALDH^+^) cells expressed higher levels of phosphorylated STAT3 compared to ALDH-negative (ALDH^−^) cells. There was a significant correlation between the nuclear staining of phosphorylated STAT3 and the expression of ALDH1 in breast cancer tissues. These results suggest that STAT3 is activated in ALDH^+^ subpopulations of breast cancer cells. STAT3 inhibitors Stattic and LLL12 inhibited STAT3 phosphorylation, reduced the ALDH^+^ subpopulation, inhibited breast cancer stem-like cell viability, and retarded tumorisphere-forming capacity *in vitro*. Similar inhibition of STAT3 phosphorylation, and breast cancer stem cell viability were observed using STAT3 ShRNA. In addition, LLL12 inhibited STAT3 downstream target gene expression and induced apoptosis in ALDH^+^ subpopulations of breast cancer cells. Furthermore, LLL12 inhibited STAT3 phosphorylation and tumor cell proliferation, induced apoptosis, and suppressed tumor growth in xenograft and mammary fat pad mouse models from ALDH^+^ breast cancer cells. Similar *in vitro* and tumor growth *in vivo* results were obtained when ALDH^+^ cells were further selected for the stem cell markers CD44^+^ and CD24^−^.

**Conclusion:**

These studies demonstrate an important role for STAT3 signaling in ALDH^+^ and ALDH^+^/CD44^+^/CD24^−^ subpopulations of breast cancer cells which may have cancer stem cell properties and suggest that pharmacologic inhibition of STAT3 represents an effective strategy to selectively target the cancer stem cell-like subpopulation.

## Introduction

Although a large number of chemotherapeutic agents have been developed which are capable of producing regression of metastatic breast cancers, these tumors usually recur following chemotherapy treatment. According to the cancer stem cell model, tumors originate in either tissue stem cells or progenitor cells through deregulation of the normally tightly regulated process of self-renewal [1], [2]. Cancer stem cells have self-renewal capacity, which drives tumorigenicity, recurrence, and metastasis. They also have the capability to differentiate, albeit aberrantly, giving rise to a heterogeneous subpopulation of constituting the tumor bulk. Recent experimental evidence suggests the existence of a small population of tumorigenic stem/progenitor cells responsible for breast tumor initiation, resistance to chemotherapy and radiation, invasion and metastasis [3]–[5]. Breast cancer cells that express the cell surface molecule CD44 (CD44^+^) but lack or have low expression of CD24 (CD24^−^) have been shown to have cancer stem cell properties [3]. More recently, an additional marker of stem/progenitor cells of breast carcinomas, aldehyde dehydrogenase 1 (ALDH1), a detoxifying enzyme responsible for the oxidation of intracellular aldehydes, was identified [4], [5]. ALDH-positive (ALDH^+^) breast cancer cells display cancer stem cells properties both *in vitro* and *in vivo,* including tumorsphere-forming capacity in anchorage-independent conditions, self-renewal, increased invasiveness, tumor-generating capacity, and metastatic potential [4]–[6]. Furthermore, in a series of 577 breast carcinomas, expression of ALDH1 correlated with poor prognosis [5].

The STAT3 protein plays a role in relaying extracellular signals initiated by cytokines and growth factors from the cytoplasm to the nucleus [7], [8]. Evidence that dysregulated STAT3 was sufficient for neoplastic transformation was provided by experiments which showed that constitutively active forms of STAT3 (phosphorylated STAT3) were capable of promoting malignant transformation in fibroblasts and tumor formation in mice [9]. In contrast, STAT3 deficient fibroblasts were shown to be resistant to transformation by a variety of oncogenes [10]. The constitutive activation of STAT3 is frequently detected in primary mammary cancer specimens as well as in established breast cancer cell lines, but not in normal mammary epithelial cells. Evidence indicates that this activation promotes tumor growth and metastasis and is crucial to the survival and growth of tumor cells [11]. Although the role of STAT3 signaling in cancer stem or cancer-initiating cells is still unknown, this pathway might represent an attractive therapeutic target. This highlights the importance of determining the role of STAT3 activation in tumor stem cell behavior as well as the effects of initiating this pathway on tumor growth. We demonstrate that the ALDH^+^ and ALDH^+^/CD44^+^/CD24^−^ subpopulations of breast cancer cells expresses higher levels of phosphorylated STAT3 (Tyrosine 705) (P-STAT3, Y705) than cell populations that do not express these stem cell markers. Furthermore, a novel STAT3 inhibitor, LLL12, suppresses ALDH^+^ and ALDH^+^/CD44^+^/CD24^−^ subpopulations of breast cancer cells *in vitro* and inhibits tumor growth in mouse xenograft and mammary fat pad models *in vivo*. These results suggest that STAT3 may represent a target for therapeutic intervention in breast cancer stem-like cells and inhibition of constitutive STAT3 signaling may provide a novel therapeutic approach.

## Materials and Methods

### Cell Culture

MDA-MB-231 and SK-BR-3 breast cancer cells were acquired from the American Type Culture Collection (Manassas, VA) and maintained in Dulbecco's Modification of Eagle's Medium supplemented with 10% fetal bovine serum (FBS) (Invitrogen). The SUM159 breast cancer cells are commercially available (Asterand, Detroit, MI). These three cancer cell lines have been routinely tested and authenticated by the American Type Culture Collection and Asterand respectively. SUM159 cells were cultured in Ham’s F12 containing 5% FBS, 5 µg/ml insulin, 1 µg/ml hydrocortisone and 10 ng/ml epidermal growth factor. ALDH^+^ and ALDH^+^/CD44^+^/CD24^−^ cells were grown in a serum-free mammary epithelial basal medium (MEBM) (Clonetics division of Cambrex BioScience) supplemented with B27 (Invitrogen), 20 ng/mL EGF (BD Biosciences), 4 ug/ml Gentamycin (Invitrogen), 1 ng/ml Hydrocortisone (Sigma-Aldrich), 5 µg/ml Insulin and 100 µM beta-mercaptoethanol (Sigma-Aldrich).

### Separation of the ALDH^+^ and ALDH^+^/CD44^+^/CD24^−^ Subpopulations of Breast Cancer Cells

The ALDEFLUOR kit (StemCell Technologies) was used to isolate the population with high ALDH enzymatic activity as previously described [5]. Briefly, cells were trypsinized to single cells using 0.05% trypsin and subsequently suspended in ALDEFLUOR assay buffer containing ALDH substrate (BAAA, 1 µmol/l per 1×10^6^ cells) and then incubated for 40 minutes at 37°C. For each sample, an aliquot of cells was stained under identical conditions with 15 mmol/L diethylaminobenzaldehyde (DEAB), a specific ALDH inhibitor, as an ALDH^−^ control. Anti-human PE-CD24 and PE-Cy5-CD44 antibody (BioLegend) were used for CD44/CD24 identification and to separate ALDH^+^/CD44^+^/CD24^−^ and ALDH^−/^CD44^+^/CD24^+^ cells when combined with ALDH staining. Analysis was performed using a FACStarPLUS (Becton Dickinion) flow cytometer. To assess the effect of STAT3 inhibitors on the subpopulation of ALDH^+^ cells, un-separated breast cancer cells were treated with 5 µmol/L of LLL12 or 10 µmol/L Stattic for 24 hours prior to performing the ALDEFLUOR assay.

### Tissue Microarray Slides, Immunohistochemistry, and Immunofluorescence Staining

Human breast cancer tissue microarray slides, comprising 95 breast cancer cases, were obtained from the Biochain Institute, Inc. [12]. These slides were baked at 60°C for 1 hour. After deparaffinization, the slides were boiled in a pressure cooker filled with 10 mM Sodium Citrate (PH6.0), and then subjected to immunohistochemistry or immunofluorescence staining. P-STAT3 (Y705) (1∶25; Signaling Technology, Beverly, MA), ALDH1 (1∶100; BD Pharmingen, San Diego, CA), Ki-67 (1∶100; Santa Cruz Biotechnology, Santa Cruz, CA) or cleaved Caspase-3 (1∶100; Signaling Technology, Beverly, MA) antibody were used. For immunofluorescence, the slides were incubated with both primary antibodies and double-stained with Alexa Fluor® 488 conjugated anti-rabbit IgG and Alexa Fluor® 594 conjugated anti-mouse IgG (Cell Signaling Technology, Beverly, MA) overnight at 4°C. The nuclei were subsequently stained with DAPI.

For immunohistochemistry, endogenous peroxidase activity was quenched by incubation in 3% hydrogen peroxide for 10 min. After blocking, the slides were incubated with primary antibody overnight at 4°C. The Histostain-Plus Kits (Invitrogen, Carlsbad, CA) were used per the manufacturer’s protocol. The slides were counterstained with hematoxylin and mounted with CRYSTAL/MOUNT (Biomeda Corp., Foster City, CA). The staining intensity was scored under microscope as described elsewhere [13]. Significance of correlation between P-STAT3 and ALDH1 was determined using two-sided Pearson Chi-square (χ^2^) test. A P-value <0.05 was considered statistically significant.

### STAT3 Inhibitors and Lentivirus Short Hairpin RNA (ShRNA)

LLL12, a STAT3 inhibitor, was synthesized in Dr. Pui-Kai Li’s laboratory. Stattic, a previously reported STAT3 inhibitor [14], was purchased from Calbiochem (San Diego, CA). ShRNA that specifically targets human STAT3 [15] and a control lentivirus that expresses Green Fluorescent Protein (GFP) were purchased from purchased from Santa Cruz Biotechnology (Santa Cruz, CA).

### Western Blot Analysis

After sorting by flow cytometry, ALDH^+^ and ALDH^+^/CD44^+^/CD24^−^ stem-like cells were cultured in serum-free stem cell medium in ultra-low attachment six-well plates (Corning) to maintain cancer stem cell characteristics. ALDH^−^, ALDH^−/^CD44^+^/CD24^+^cells and un-separated cells were cultured in regular medium and replaced with identical stem cell medium for three days before being harvested. To assess the effects of STAT3 inhibitors, ALDH^+^ and ALDH^+^/CD44^+^/CD24^−^ subpopulations of breast cancer cells were treated with LLL12 (5 µmol/L) of or static (10 µmol/L) for 24 hours. STAT3, or control GFP shRNA lentivirus (CTL ShRNA) was introduced into ALDH^+^ breast cancer stem-like cells for 48 hours, then followed by selection with puromycin (0.2 µg/ml) for 72 hours. Antibodies (Cell Signaling Tech.) against phospho-specific STAT3 (Tyrosine 705) (P-STAT3, Y705), ERK1/2 (Threonine 202/Tyrosine 204), cleaved Poly (ADP-ribose) polymerase (PARP), ALDH1, cleaved caspase-3, and GAPDH were used for western blots.

### Reverse Transcriptase-Polymerase Chain Reaction (RT-PCR)

The ALDH^+^ and ALDH^+^/CD44^+^/CD24^−^ subpopulations of breast cancer cells were treated with LLL12 (5 µM) or DMSO for 24 h. RNA from the cells was then collected using RNeasy Kits (Qiagen). cDNA was constructed from a 500 ng sample of RNA using Omniscript RT (Qiagen). Primer sequences and source information can be found in Table S1. The intensity of bands was quantified and normalized to GAPDH.

### Tumorsphere Culture

ALDH^+^ and ALDH^+^/CD44^+^/CD24^−^ cells were plated as single cells in ultra-low attachment six-well plates at a density of 250, 500, 1000 or 50,000 viable cells/well as previously described [4]. On the second day after seeding, the ALDH^+^ cancer cells were treated with 2.5–10 µmol/L of LLL12 or Stattic. For tumorsphere forming capacity assay, ALDH^+^ or ALDH^−^ cells were plated as single cells in ultra-low attachment six-well plates at a density of 250, 500 or 1000 viable cells/well. Tumorsphere growth was observed under a microscope 10 to 15 days later.

### Kinase Activity Assay

The effects of LLL12 on twenty six purified human protein kinases were performed at the Millipore UK Limited (Dundee, UK) and Reaction Biology Corp. (Malvern, PA) using the Kinase profiler assay. Assays contained a peptide substrate, purified recombinant human protein kinases to be tested, and gamma-labeled ATP, magnesiumion. Radioactive phosphorylated product was measured and quantitated via a scintillation counter. Appropriate kinase inhibitor, which gave half-maximal inhibitory concentrations (IC50) values at nM ranges was used as a positive control. The IC50 inhibitory values of LLL12 on the kinase activity were determined using 10 different concentrations of LLL12 with 100 µM as the highest concentration.

### MTT Cell Viability Assay

The breast cancer cells were seeded in 96-well plates (3,000 cells/well) in triplicates in a serum-free mammary epithelial basal medium. The following day, cancer cells were treated with 1 to 10 µmol/L of LLL12 or Stattic for 72 hours, STAT3 ShRNA or control GFP shRNA lentivirus (CTL ShRNA) for 48 hours. MTT (Thiazolyl Blue Tetrazolium Bromide, Sigma-Aldrich) assay was used to determine the cell viability.

### Mouse Xenograft and Orthotopic Tumor Models

All animal studies were conducted in accordance with the principles and standard procedures approved by IACUC at the Research Institute at Nationwide Children’s Hospital and the University Committee on the Use and Care of Animals at the University of Michigan. ALDH^+^ subpopulations of MDA-MB-231 and ALDH^+^/CD44^+^/CD24^−^ subpopulations of SUM-159 breast cancer cells (1×10^5^) respectively were injected *subcutaneous (s.c.)* into the flank area of female Non-obese diabetic (NOD)/Severe combined immunodeficiency (SCID) mice which were purchased from Jackson Laboratory.

For mammary fat pad experiments, 1×10^5^ of sorted SUM159 ALDH^+^ cells, assessed using Aldefluor kit (StemCell Technologies) were injected into the humanized fat pads of NOD/SCID mice (The Jackson Laboratory), as previously described [5]. After tumor development, mice were randomly divided into two treatment groups consisting of 5–6 mice/group, and treated with vehicle control or 5 mg/kg of LLL12 (dissolved in 10% DMSO, 18% Cremophor EL and 72% sterile 5% Dextrose) via *intraperitoneal (IP)* daily. For ShRNA lentivirus study, after sorting ALDH^+^ MDA-MB-231 cancer stem-cell like cells (1×10^5^) were infected with STAT3, or GFP shRNA lentivirus (CTL ShRNA) for 48 hours. After 72 hours of selection with puromycin, cells were mixed with an equal volume of Matrigel and injected subcutaneously into the flanks area of 4- to 5-week-old female NOD/SCID mice. Tumors were measured by a caliper and the volume was calculated using *V* = π (width^2^×length)/6. After 15 days of treatments with LLL12 or vehicle control, tumors were harvested from euthanized mice, snap-frozen in liquid nitrogen, and stored in −80°C. A portion of tumor tissues was embedded in Tissue-Tek OCT compound (Miles, IN), and stored at −80°C until use for IHC staining. The rest of tumors tissues were homogenized to examine the expression of STAT3 phosphorylation by western blot.

## Results

### The ALDH^+^ Subpopulation of Breast Cancer Cells Expresses High Levels of STAT3 Phosphorylation

**Figure 1:**
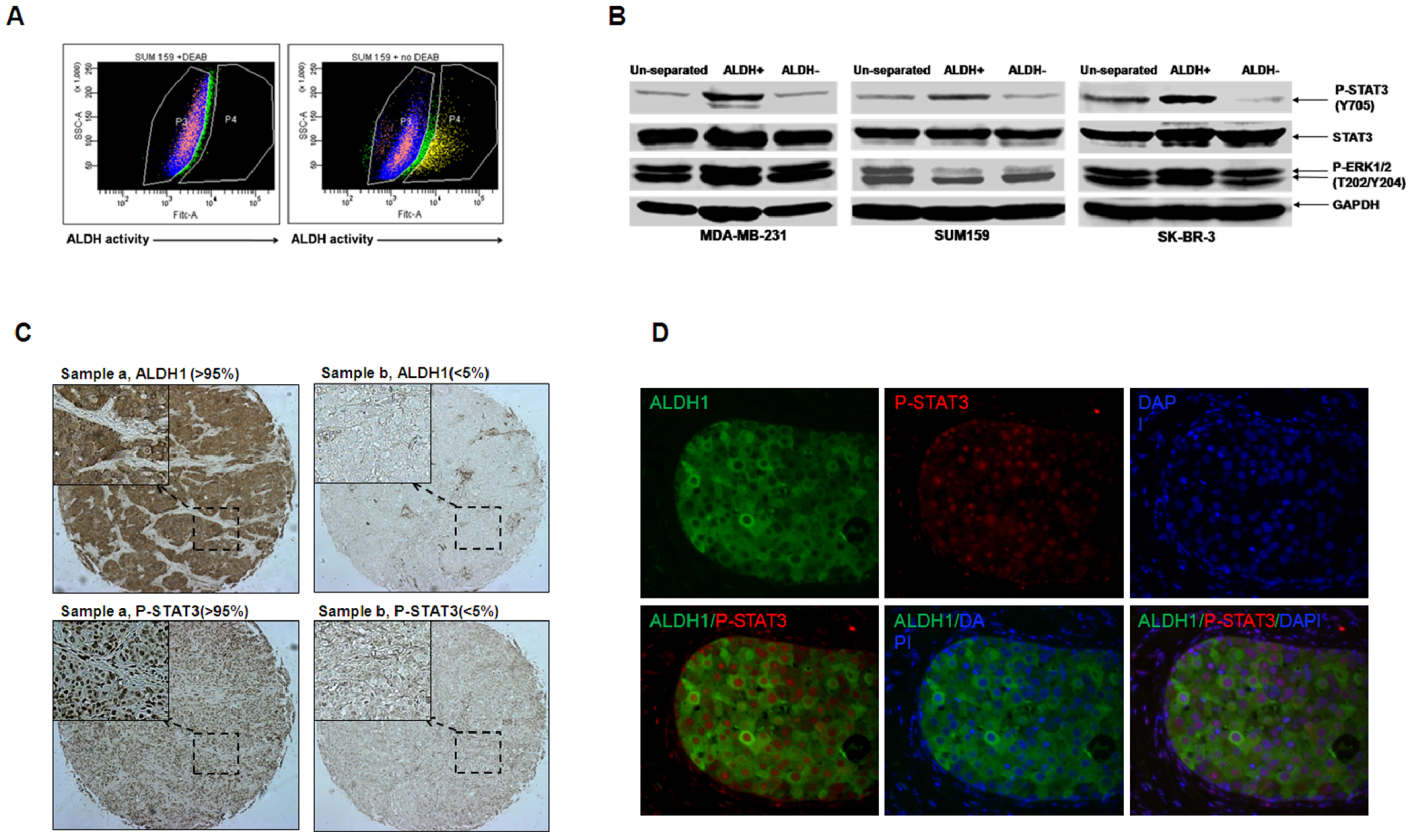
STAT3 phosphorylation of ALDH^+^ subpopulation of breast cancer cells was higher than un-separated and ALDH^−^ subpopulations. (**A**) Representative flow cytometry analysis of ALDH enzymatic activity in SUM159 breast cancer cells was shown. (**B**) ALDH^+^ and ALDH^−^ subpopulations were separated from MDA-MB-231, SUM159, and SK-BR-3 breast cancer cells by flow cytometry. Phosphorylation of STAT3 (Y705), and ERK 1/2 (T202/Y204), was detected by Western blot. (**C**) Breast cancer tissue microarray slides were stained using immunohistochemistry (IHC). Representative examples of ALDH1 positive/P-STAT3 (Y705) positive (>95%, sample a) and ALDH1 negative/P-STAT3 (Y705 negative (<5%, sample b) tumors were shown. The spots for ALDH1 and P-STAT3 (Y705) were from the matched tissues section from the same patient. (**D**) Breast cancer tissue microarray slides were double-stained with P-STAT3 and ALDH1. Representative examples of the expression of STAT3 phosphorylation and ALDH1 was shown by immufluorence (IF) staining. ALDH1 high expression tumor cells (cytoplasm, green) also expressed P-STAT3 in nuclear (red).

To determine the expression of the activated P-STAT3 in breast cancer stem cells, we separated the ALDH^+^ and ALDH^−^ subpopulations of three breast cancer cell lines, MDA-MB-231, SUM159, SK-BR-3 and determined the level of P-STAT3 by Western blot. A representative example of using flow cytometer to separate ALDH^+^ cells in SUM159 is shown in Figure 1A. It has been demonstrated that the ALDH^+^ (but not the ALDH^−^) subpopulations of these breast cancer cells exhibit cancer stem cell properties *in vitro* and in the mouse tumor xenographs [4]–[6]. To confirm the cancer stem cell properties of ALDH^+^ subpopulations, we compared the tumorspere-forming ability between ALDH^+^ and ALDH^−^ subpopulations. As shown in Table 1, ALDH^+^ cells from SUM159, MDA-MB-231, and SKBr3 breast cancer cells all generated more tumorsperes than ALDH^−^ cells. Our results showed that ALDH^+^ subpopulation of breast cancer cells expressed higher levels of P-STAT3 (Y705) compared to un-separated or ALDH^−^ cells, with the latter subpopulation displaying the lowest level of P-STAT3 (Figure 1B). Phosphorylation at Tyrosine residue 705 (Y705) is important for STAT3 activation [16]. In contrast to differences in STAT3 phosphorylation, ERK1/2 phosphorylation at threonine 202/tyrosine 204 (T202/Y204) was not consistently high in the ALDH^+^ subpopulation. These results suggest that ERK likely does not play a key role in ALDH^+^ breast cancer stem-like cells. The increase in expression of phosphorylated STAT3 in the ALDH^+^ subpopulation suggests a possible role for this pathway in breast cancer stem-like cells.

**Table 1:** ALDH^+^ and ALDH^−^ subpopulations of breast cancer cells were separated by flow cytometer and cultured in stem cell medium as described in Materials and Methods.

		SUM159	MDA-MB-231	SKBr3
250 cells/well	**ALDH+**	2.7±1.5*	15.7±1.5*	12.7±1.5*
	**ALDH**−	**0**	**4.3±1.5**	**4.7±1.5**
500 cells/well	**ALDH+**	20±4.9*	27.7±4.7*	14.7±1.2*
	**ALDH**−	**3±2**	**6±6.1**	**7.3±1.5**
1000 cells/well	**ALDH+**	27±2.6*	46±9.5*	23.3±4*
	**ALDH**−	**7±2.6**	**27.7±3.1**	**9.3±2.1**

The numbers of tumosphere generated per 250, 500 or 1000 cells were counted two weeks later. (*p<0.05 vs ALDH−).

To determine whether similar association of STAT3 phosphorylation was also present in primary human breast cancer samples, we examined the relationship between P-STAT3 (Y705) and ALDH1 protein expression in human breast cancer tissues using tissue microarrays. We observed a significant association (P<0.05) between expression of nuclear P-STAT3 and ALDH1 (Table 2). However, we did not detect significant association of double positive of P-STAT3 and ALDH1 with the clinical or pathological features such as tumor size, histological type, grade, lymph node or distant Metastasis, AR, ER, PR, and HER2 (Table S2). This may be due to our sample’s numbers are not big enough. Representative examples of immunohistochemistry/immunofluorescence staining of P-STAT3 and ALDH1 are shown in Figure 1C and 1D. The data obtained from breast cancer patient samples and the data from the cell lines all demonstrated that increased expression of P-STAT3 might correlated the expression or activity of ALDH. Furthermore, if the activation of STAT3 plays a role in breast cancer stem-like cells then inhibition of this pathway represents a rational strategy to target the breast cancer stem cell-like populations.

**Table 2:** The correlation of P-STAT3 (Y705) with ALDH1 (Both positive or negative) was assessed by χ^2^ Test*.

Number (%)		ALDH1		χ^2^	P*
		Positive	Negative		
P-STAT3	Positive	18 (18.94%)	15 (15.79%)	25.358	4.8e-7
	Negative	5 (5.26%)	57 (60.0%)		

*P*<0.05 is considered as statistical significance. Breast cancer tissues from a total numbers (n) of 95 cancer patients were examined.

### LLL12, a Small Molecular STAT3 Inhibitor, Selectively Inhibits STAT3 Phosphorylation, STAT3 Downstream Targets, and Induces Apoptosis in Breast Cancer Cells

To confirm the importance of STAT3 in breast cancer stem-like cells, the STAT3 inhibitor, LLL12 [17] (Figure S1), which is a novel analog of a previously reported STAT3 inhibitor LLL3 [18], was used to target STAT3 in breast cancer stem-like cells. LLL12 contacts the STAT3 SH2 domain at Y705 and partially binds to the side pocket close to Y705 in a computer docking model via AutoDock. To confirm the inhibition of STAT3, we examined the effects of LLL12 on STAT3 phosphorylation in three independent breast cancer cell lines. Our results demonstrated that LLL12 inhibited STAT3 phosphorylation, expression of STAT3 target genes including Cyclin D1, survivin [19], Bcl-2 [9] and Twist1 [20], and subsequently induced apoptosis as indicated by an increase in levels of cleaved PARP and Caspase-3 in MDA-MB-231, SK-BR-3, and SUM159 breast cancer cell lines (Figure S2). The specificity of inhibition was demonstrated by the observation that LLL12 did not inhibit the phosphorylation of ERK. In addition, LLL12 exhibited little inhibition (IC50 are greater than 100 µM) on the tyrosine kinases, Fes, JAK2, Bmx, c-SRC, PYK2, Syk, Fyn, and Yes containing SH2 domains or both SH2 and SH3 domains (Table S3). LLL12 also produced little inhibition (IC50 are 77.94 µM or greater) of other protein kinases that are involved in cell proliferation and survival including AKT1, c-Raf, EGFR, ErB2/HER2, Met, mTOR, PDK1, PI3K, and others (Table S3). Positive controls for these kinase assays including PI3K inhibitor, LY294002 (IC_50_ is 0.785 and 0.243 µM on PI3Kα and PI3Kβ respectively), P38 inhibitor, SB202190 (IC_50_ is 0.011 µM on P38), and Staurosporine (IC_50_ between <0.001 and 0.456 µM). LLL12 also inhibited STAT3, but not STAT1 DNA binding activity [17]. These results strongly support the specificity of LLL12 in the inhibition of STAT3 and suggest it may be a useful agent to target breast cancer stem-like cells.

### LLL12 Inhibits STAT3 Phosphorylation and STAT3 Pathway in Downstream Targets in ALDH^+^ Cells

**Figure 2:**
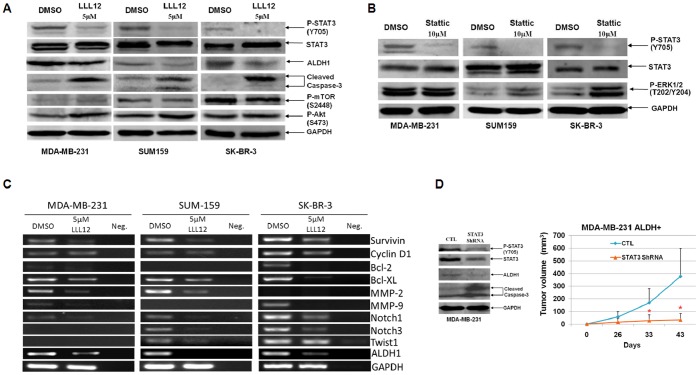
LLL12 and Stattic inhibited STAT3 expression. (**A**) LLL12 inhibited STAT3 phosphorylation and induced apoptosis in ALDH^+^ breast cancer stem-like cells. (**B**) Stattic inhibited STAT3 but not ERK1/2 phosphorylation in ALDH^+^ breast cancer stem-like cells. (**C**) LLL12 inhibited the expression of STAT3 downstream target genes and ALDH1 in ALDH^+^ subpopulation of breast cancer cells. (**D**) STAT3 ShRNA decreased the STAT3 expression and STAT3 phosphorylation, and inhibited tumor growth in ALDH^+^ MDA-MB-231 breast cancer stem-like cells.

We next examined the effect of LLL12 on breast cancer stem-like cells. LLL12 inhibited STAT3 phosphorylation and induced cleaved caspase-3 in the ALDH^+^ subpopulation of MDA-MB-231, SUM159, and SK-BR-3 cells (Figure 2A). There was almost no effect on mTOR and AKT phosphorylation in all three cell lines (Figure 2A). Another previously reported STAT3 inhibitor Stattic [14] also reduced P-STAT3 expression in ALDH^+^ cells (Figure 2B). The inhibition of STAT3 by LLL12 also down-regulated the expression of known STAT3 target genes in ALDH^+^ breast cancer cells related to cancer cell proliferation, survival, and angiogenesis, including Cyclin D1, survivin [19], Bcl-2, Bcl-XL [9], MMP-2, and MMP-9 [21], [22] (Figure 2C). Furthermore, LLL12 inhibited Twist1 [20], Notch-1, and Notch-3 [23] expression in ALDH^+^ breast cancer stem-like cells, which have recently been reported as STAT3 or Interleukin-6 target genes (Figure 2C). The inhibition of LLL12 on these STAT3 target genes in ALDH^+^ stem cell-like breast cancer cells was quantified and normalized to GAPDH (Table S4). Twist1 has been shown to play an important role in the epithelial to mesenchymal transition and malignant transformation [20]. The Notch signaling pathway is known to be essential for normal stem cell self-renewal and differentiation in a variety of tissues, and is involved in human cancer stem cells’ self-renewal capacity and tumorigenicity [23], [24].

To verify that the effects of LLL12 were due to STAT3 inhibition we determined whether similar effects resulted from STAT3 inhibition by ShRNA. We observed that STAT3 ShRNA down regulated STAT3 expression and phosphorylation, and induced the cleavage of caspase-3 in ALDH^+^ breast cancer cells. STAT3 shRNA significantly suppressed ALDH^+^ breast cancer stem-like cell tumor growth compared with lentivirus GFP as a control (Figure 2D). LLL12 and STAT3 ShRNA also down-regulated the expression of ALDH1 protein (Figure 2A, 2C, 2D).

### STAT3 Inhibitors, LLL12 and Stattic Reduce the ALDH^+^ Subpopulation of Breast Cancer Cells

**Figure 3:**
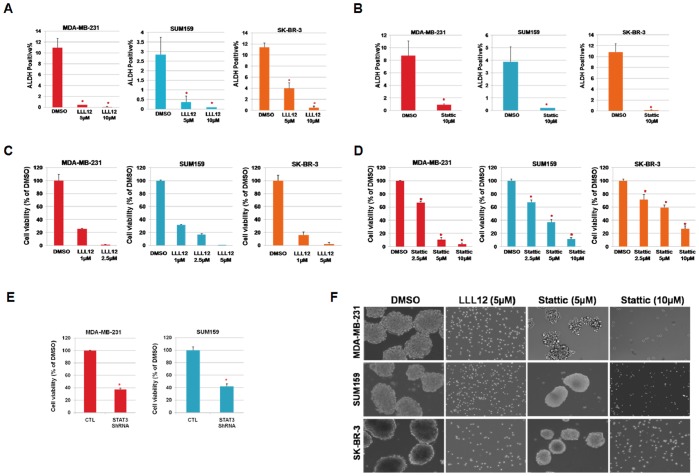
LLL12, Stattic and STAT3 ShRNA inhibited ALDH^+^ cell viability. LLL12 (**A**) and Stattic (**B**) reduced the ALDH^+^ subpopulation of MDA-MB-231, SUM159, and SK-BR-3 breast cancer cells. Statistically significant reduction of LLL12-treated relative to the DMSO is designated by an asterisk (*P*<0.05). LLL12 (**C**), Stattic (**D**), and STAT3 ShRNA (**E**) inhibited cell viability of ALDH^+^ subpopulation of breast cancer cells. CTL: control lentivirus that expresses GFP. (**F**) LLL12 and Stattic inhibited tumorsphere formation of the ALDH^+^ subpopulation of breast cancer cells.

Cancer stem cells are relatively resistant to radiation and chemotherapy [25], [26]. To examine whether LLL12 might effectively target the ALDH^+^ subpopulation, we determined the effect of this STAT3 inhibitor on the percentage of ALDH expression in breast cancer cells. LLL12 treatment resulted in a decrease in the ALDH^+^ subpopulation in MDA-MB-231, SUM159, and SK-BR3 cancer cells (Figure 3A), suggesting that this subpopulation of breast cancer stem-like cells is more sensitive to LLL12-mediated inhibition than is the bulk cell population. A representative example of flow cytometry analysis of ALDH^+^ cells in SUM159 breast cancer cells treated with LLL12 is shown in Figure S3. We found that 10 µM of Stattic, another previously reported STAT3 inhibitor [14], also decreased the percentage of ALDH^+^ subpopulation (Figure 3B). These results provide evidence that STAT3 signaling plays an important role in maintenance of the ALDH^+^ population.

### ALDH^+^ Cells are Sensitive to the Inhibition by STAT3 Inhibitors and STAT3 ShRNA

We found that LLL12 (Figure 3C) and Stattic (Figure 3D) could inhibit cell viability of the ALDH^+^ subpopulation from MDA-MB-231, SUM159, and SK-BR3 cells, although LLL12 is more potent than Stattic in terms of inhibiting breast cancer initiating cell viability. Also, STAT3 ShRNA reduced cell viability of the ALDH^+^ cells (Figure 3E). Taken together, these results support that breast cancer stem-like cells are sensitive to STAT3 inhibitors. The possible effects of the STAT3 inhibitors and ShRNA on ALDH^−^ cells were also examined. We observed some inhibitory effects against the ALDH^−^ cells following treatment with LLL12, Stattic and STAT3 ShRNA (Figure S4A–C). This may be expected, however, as ALDH^−^ cells still express a low level of STAT3 phosphorylation (Figure 1B).

One of the hallmarks of mammary tumor stem and progenitor cells is their ability to survive and proliferate in anchorage-independent conditions and form floating spherical colonies known as “tumorspheres” [4], [5], [25], [27]. We were able to suppress tumorsphere formation by the ALDH^+^ subpopulations of SK-BR-3, MDA-MB-231, and SUM159 using LLL12 and Stattic (Figure 3F). Again, LLL12 was more potent than Stattic in inhibiting tumorsphere formation. The enhanced efficacy of LLL12 may be explained by its higher predicted binding affinity for STAT3. In a computer model, LLL12 had higher binding affinity (−7.8 Kcal/mol) than Stattic (−5.6 Kcal/mol) for the STAT3 SH2 domain, a difference of 57.8-fold.

### LLL12 Suppresses Tumor Growth from Breast Cancer Stem-like Cells in Mouse Tumor Xenografts and an Orthotopic Model in vivo

**Figure 4:**
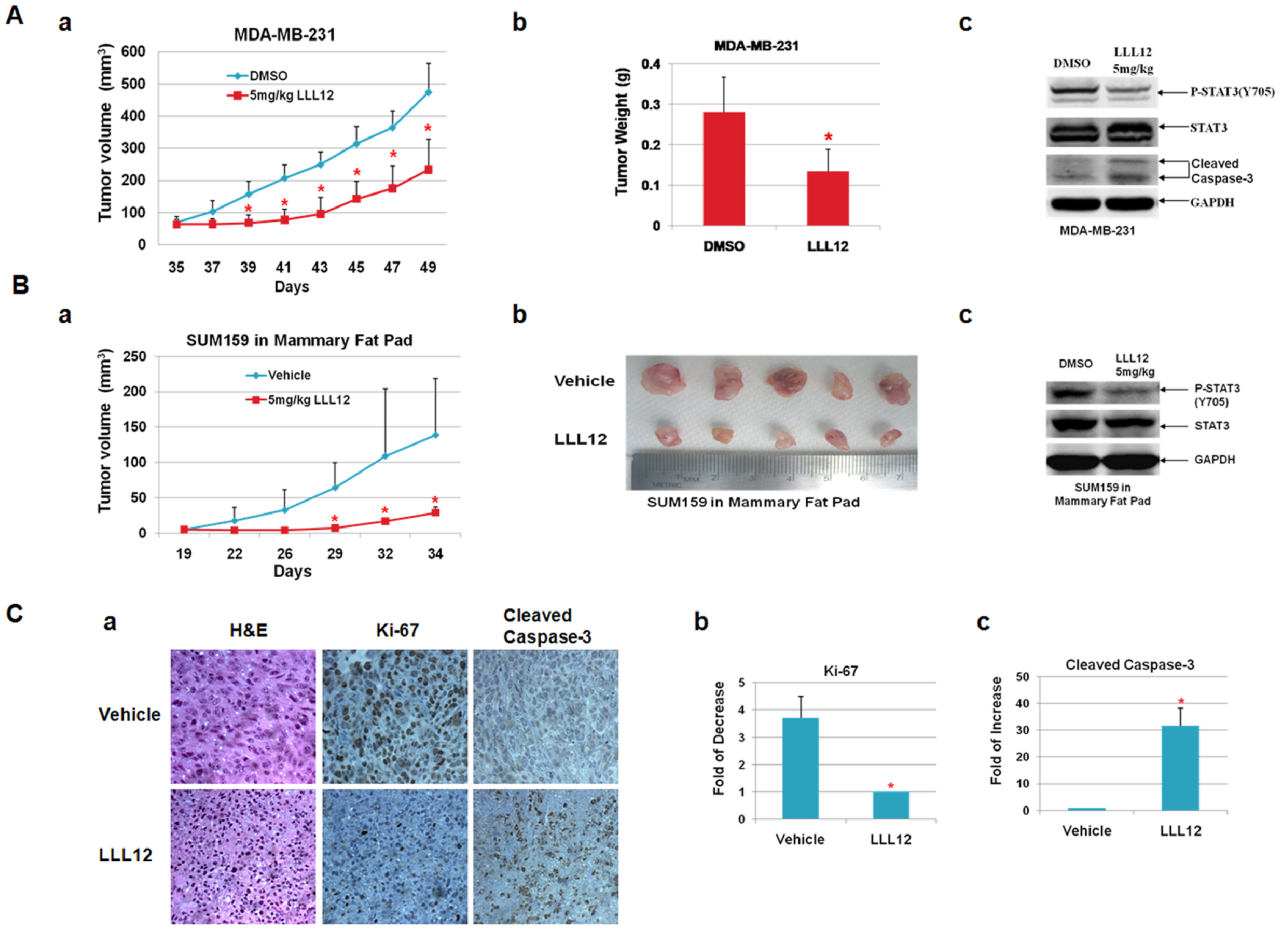
LLL12 suppressed tumor growth. LLL12 suppressed tumor growth in (**A**) mouse xenografts with MDA-MB-231 breast cancer stem-like cells and (**B**) mammary fat pad with SUM159 breast cancer stem-like cells (ALDH^+^ cells). Reduction of tumor volume (**Aa** and **Ba**) and tumor weight (**Ab** and **Bb**) in all LLL12-treated mice compared to vehicle group (*P<0.05). One representative sample from tumor tissues generated from ALDH^+^ MDA-MB-231 cancer stem-like showing STAT3 phosphorylation were also inhibited by LLL12 treatment (**Ac** and **Bc**). (**C**) Immunohistochemistry staining of tumor xenografts was performed using Ki-67 and cleaved caspase-3 antibodies. The staining was visualized and photographed on a BX51 fluorescence microscope (Olympus, Tokyo, Japan) at x200 magnification (**Ca**). Positively stained cells in each photo were counted. LLL12 decreased the number of Ki-67 positive tumor cells (**Cb**) and increased the numbers of cleaved caspase-3 positive tumor cells (**Cc**).

To determine whether LLL12 may have a therapeutic potential, we determined the effects of systemic administration of LLL12 on tumor growth in NOD/SCID mice. Our results showed that LLL12 significantly suppressed (*P*<0.05) tumor volume (Figure 4A–a), tumor weight (Figure 4A–b) of MDA-MB-231 ALDH^+^ breast cancer stem-like cells in the xenograft mouse model. This was accompanied by decreased express of P-STAT3 and increased cleaved caspase-3 (Figure 4A–c).

Similar results were observed in the mouse mammary fat pad model with SUM159 ALDH^+^ breast cancer stem-like cells. Treatment of these mice with LLL12 resulted in significant suppresses (*P*<0.05) of tumor volume (Figure 4B–a) and tumor mass (Figure 4B–b), and STAT3 phosphorylation (Figure 4B–c) in SUM159 breast cancer stem-like cells. In addition, we examined Ki-67 and cleaved caspase-3 expression by immunohistochemistry staining in tumor tissues from breast cancer stem-like cells (Figure 4C). The results showed that STAT3 inhibitor, LLL12 decreased number of Ki-67 positive cells (Figure 4C–b) and increased number of cleaved caspase-3 positive cells (Figure 4C–c). These data indicated that LLL12 inhibited proliferation and induced apoptosis in tumors. Body weight did not differ in LLL12 treated compared to a vehicle control (data not shown) indicating a lack of systemic toxicity of LLL12. These results demonstrate that LLL12 is potent in inhibition of STAT3 signaling with a resultant decrease in the ALDH^+^ cell population and tumor growth.

### ALDH^+^/CD44^+^/CD24^−^ Subpopulation of Breast Cancer Cells also Expresses High Levels of STAT3 Phosphorylation and is Sensitive to LLL12 Inhibition

**Figure 5:**
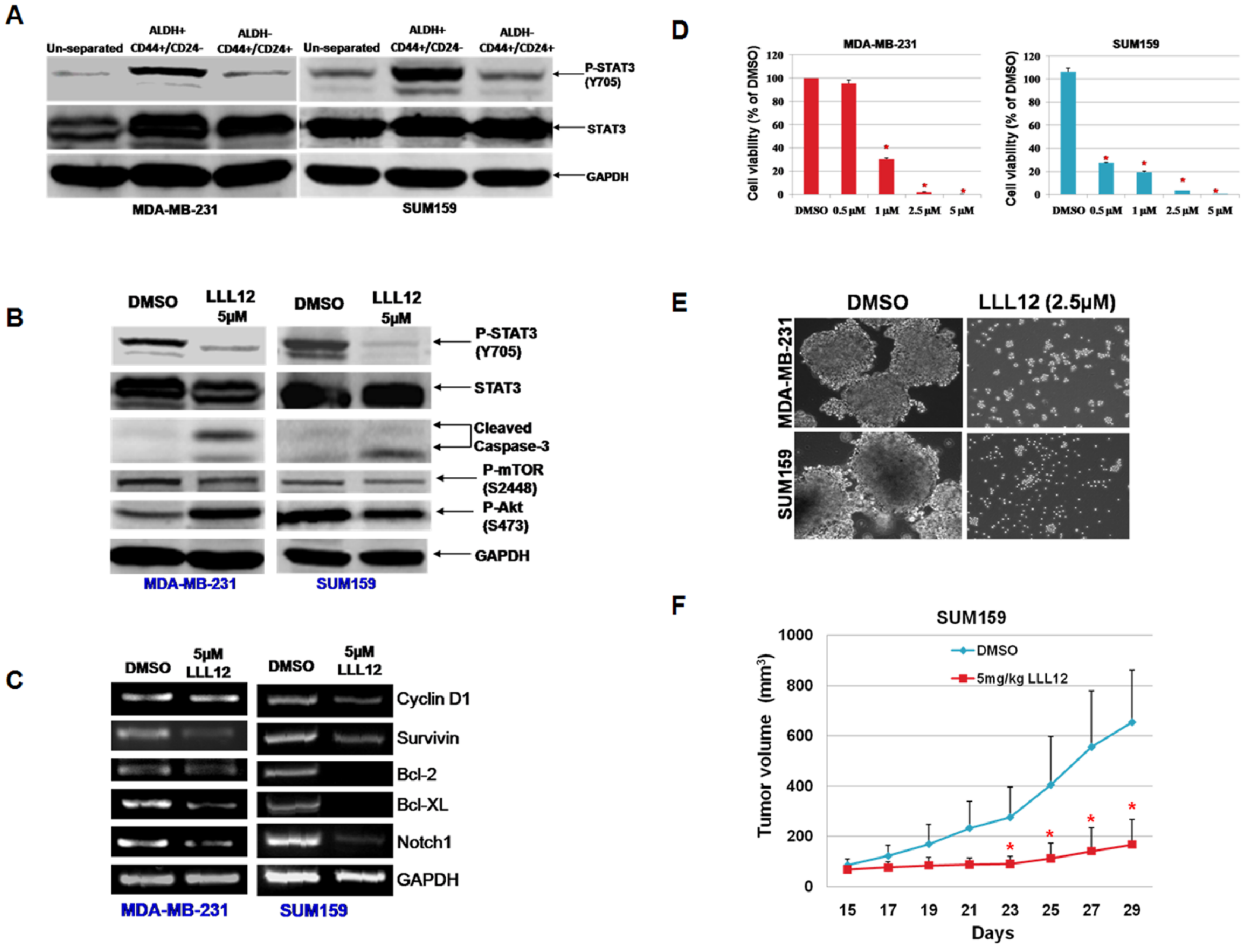
LLL12 inhibited ALDH^+^/CD44^+^/CD24^−^ subpopulations in vitro and in vivo. ALDH^+^/CD44^+^/CD24^−^ and ALDH^−/^CD44^+^/CD24^+^ subpopulations were separated from MDA-MB-231 and SUM159 breast cancer cells by flow cytometry. (**A**) STAT3 phosphorylation of the ALDH^+^/CD44^+^/CD24^−^ subpopulation of breast cancer cells was higher than un-separated and the ALDH^−/^CD44^+^/CD24^+^ subpopulations. ALDH^+^/CD44^+^/CD24^−^ breast cancer stem-like cells were treated with then 0.5–5 µM of LLL12 or DMSO as indicated. LLL12 inhibited STAT3 phosphorylation, induced apoptosis (**B**) and reduced STAT3 downstream target genes expression in ALDH^+^/CD44^+^/CD24^−^ breast cancer stem-like cells (**C**). LLL12 also inhibited cell viability (**D**) and tumorsphere formation (**E**) of ALDH^+^/CD44^+^/CD24^−^ subpopulation of breast cancer cells. (**F**) LLL12 suppressed tumor growth in mouse xenografts with ALDH^+^/CD44^+^/CD24^−^ SUM-159 breast cancer stem-like cells (*P<0.05).

Breast cancer stem cells have also been reported to express the phenotype CD44^+^/CD24^−^. These markers identify overlapping but not identical breast cancer stem cell populations. Furthermore, breast cancer cells with the overlapping profile ALDH^+^/CD44^+^/CD24^−^ (Figure S5) are more enriched in tumor initiating cells than each of these markers alone. We, therefore, examined the effects of STAT3 inhibition on this more highly enriched population of breast cancer stem-like cells. The ALDH^+^/CD44^+^/CD24^−^ subpopulation of MDA-MB-231 and SUM159 breast cancer cells expressed higher levels of P-STAT3 compared to the un-separated or ALDH^−/^CD44^+^/CD24^+^ subpopulations (Figure 5A). LLL12 also inhibited STAT3 phosphorylation and induced caspase-3 cleavage in the ALDH^+/^CD44^+^/CD24^−^ subpopulation of MDA-MB-231 and SUM159 (Figure 5B). The inhibition of STAT3 by LLL12 also down-regulated the expression of several known STAT3-regulated genes in breast cancer stem-like cells such as Cyclin D1, survivin [19], Bcl-2, Bcl-XL [9] and an IL-6 regulated gene, Notch1 [23] (Figure 5C). In addition, we observed that LLL12 inhibited cell viability (Figure 5D) and tumorsphere forming capacity (Figure 5E) in the ALDH^+^/CD44^+^/CD24^+^ subpopulation of MDA-MB-231 and SUM159 breast cancer cells. We further tested LLL12 against ALDH^+^/CD44^+^/CD24^−^ breast cancer stem-like cells isolated from SUM159 cancer cells in a NOD/SCID mouse xenograft model. The results showed that LLL12 significantly suppressed (*P*<0.05) the tumor volume of SUM159 breast cancer stem-like cells (Figure 5F). These results further demonstrated that LLL12 is potent in suppressing tumor growth initiated from the breast cancer stem-like cells *in vivo*.

## Discussion

STAT3 is regulated by a wide variety of growth factors and cytokines [7], [8]. STAT3 is frequently activated in many types of human solid and blood cancers, including breast, cancer and contributes to cancer progression [11], [28]. Previous studies have focused on the effects of STAT3 inhibition on the bulk tumor cell population. Recently STAT3 has been reported to play a role in glioblastoma stem cells [29], [30]. Utilizing markers of breast cancer stem cells we demonstrate that the ALDH^+^ and ALDH^+^/CD44^+^/CD24^−^ subpopulations of breast cancer cells express higher levels of P-STAT3 (Y705) compared to the un-separated, ALDH^−^, or ALDH^−/^CD44^+^/CD24^−^ subpopulations. Our results also showed that there is a significant correlation between the nuclear staining of P-STAT3 and the expression of ALDH1 in the cancer tissue obtained from breast cancer patients. These results suggest that the STAT3 pathway may provide novel therapeutic target in breast cancer stem-like cells. To explore this we examined the inhibitory effects of two STAT3 inhibitors, LLL12 and Stattic as well as STAT3 ShRNA on breast tumor initiating cells. LLL12 is a novel and more potent derivative of LLL3 [18]. Our results showed that LLL12 is potent in inhibiting STAT3 phosphorylation, cell viability, the formation of tumorspheres, and inducing apoptosis in the ALDH^+^ subpopulation of breast cancer cells. Although stattic can also inhibit cell viability and the formation of tumorspheres in the ALDH^+^ subpopulation of breast cancer cells it is less potent than LLL12, an observation which is consistent with weaker predictive binding affinity to STAT3 than LLL12. In addition, STAT3 ShRNA also inhibits STAT3 phosphorylation and cell viability in ALDH^+^ cells *in vitro*. LLL12 can also down-regulate putative STAT3 or IL-6 downstream target genes that are involved in stem cell growth and survival including Notch 1 and Notch 3 [23] as well as known STAT3 downstream target genes, such as Cyclin D1, survivin, Bcl-2, Bcl-XL, MMP-2, and MMP-9 that are involved in cell proliferation and survival. These results determined that a novel molecular pathway, STAT3 signaling, is linked to breast cancer stem cell growth and survival. Our data also provide a possible molecular mechanism of LLL12-mediated inhibition of breast cancer stem-like cells. The observation that treatment with STAT3 inhibitors resulted in a decrease in the percent of ALDH^+^ cells suggests that ALDH^+^ cells are dependent on STAT3 signaling. The decreased percentage of ALDH^+^ positive cells may either due to inhibition of ALDH enzyme activity or selective elimination of ALDH^+^ cells or both. The ability of STAT3 inhibitors to induce apoptosis in both cancer stem cell and bulk tumor populations makes them potentially attractive therapeutic agents.

Our results also suggest that constitutively active STAT3 in these cancer stem-like cells enhances tumor growth in mice, whereas STAT3 blockade by LLL12 directly suppresses MDA-MB-231 and SUM-159 ALDH^+^ cell growth in xenograft and mammary fat pad mouse models respectively *in vivo*. Furthermore, LLL12 also suppresses the SUM-159 ALDH^+^/CD44^+^/CD24^−^ cell growth in a mouse xenograft tumor model. These *in vivo* results were consistent with the *in vitro* cancer stem cell data using LLL12, indicating that LLL12 is a potent STAT3 inhibitor in the suppression of tumor growth of breast cancer stem-like cells in the mouse model *in vivo*. Intratumor heterogeneity is a major clinical problem because tumor cell subpopulations may display variable sensitivity to therapeutics. Overall, ALDH^+^ and CD44^+^/CD24^−^ cells are more frequent in basal-like/mesenchymal and basal-like breast cancer respectively, whereas luminal tumors are enriched in ALDH^−^ and CD44^+^/CD24^−^ cells [4], [31]. Thus, therapies eliminating ALDH^+^, CD44^+^/CD24^−^ and ALDH^+^/CD44^+^/CD24^−^ cells may represent a new approach for the clinical management of triple-negative basal-like mesenchymal breast cancer, currently the only major breast tumor subtype without effective targeted treatment strategies and with poor prognosis [32]. Due to its ability to target breast cancer stem-like cells and bulk MDA-MB-231 (triple-negative, basal-like) and SUM-159 (triple-negative, mesenchymal) breast cancer cells from ALDH^+^ and ALDH^+^/CD44^+^/CD24^−^ tumor initiating subpopulation, LLL12 is a therapeutic candidate.

In summary, this study demonstrates that STAT3 is activated in ALDH^+^ and ALDH^+^/CD44^+^/CD24^−^ breast cancer cells. This is consistent with recent results demonstrating that the cytokine IL-6 is able to stimulate breast cancer stem cells [33] and JAK2/STAT3 signaling pathway is required for growth of breast cancer stem cells [34]. In addition, Stat3 was identified as a critical node in self-renewal in breast cancer tumor-initiating cells [35]. In the same paper, authors also showed that a small molecule Stat3 inhibitor could reduce breast cancer tumor-initiating cells and improve recurrence free survival in a human-xenograft model [35]. Our data also demonstrate an important role of constitutive STAT3 signaling in breast cancer stem-like cell growth *in vitro* and in mouse tumor models *in vivo*. Recent results from glioblastoma studies also showed that the IL-6/STAT3 pathway is required for proliferation, survival and tumor growth of glioblastoma stem cells [29], [30], [36]. It would be of interests to examine whether STAT3 is also activated in cancer stem cells from other types of human cancer. We show that the pharmacologic targeting of STAT3 is able to suppress ALDH^+^ and ALDH^+^/CD44^+^/CD24^−^ cells *in vitro* and in mouse tumor models. These results suggest that targeting STAT3 signaling may be useful as a cancer stem cell directed therapy in breast cancer. However, whether STAT3 inhibition can improve our success in treating breast cancer remains to be studied in future studies. In addition, STAT5 and STAT3 mediate opposing effects on several key target genes in breast cancer cells, with STAT5 exerting a dominant role [37]. It might be interested to examine what is the status of STAT5 in breast cancer stem-like cells.

## Supporting Information

Figure S1 **The synthesis of LLL12.**
(JPG)

Figure S2 **LLL12 inhibited STAT3 phosphorylation, and down-regulated STAT3-regulated genes, Cyclin D1, Survivin, Bcl-2 and Twist1, as well as induced apoptosis in un-seperated MDA-MB-231, SK-BR-3, and SUM159 breast cancer cells.** LLL12 was synthesized in Dr. Pui-Kai Li’s laboratory (College of Pharmacy, The Ohio State University). Un-separated cells were treated with 10 µM of LLL12 or DMSO for 24 hours, and the phosphorylation of STAT3 (Y705), and ERK 1/2 (T202/Y204), and expression of STAT3 downstream genes, cleaved caspase-3, and PARP were detected by Western blots.
(JPG)

Figure S3 **LLL12 (10 µM) decreased the percentage of ALDH^+^ subpopulation in SUM159 breast cancer cells.** A representative example of flow cytometry analysis of ALDH^+^ cells in SUM159 breast cancer cells treated with LLL12. ALDH^+^ (P5, yellow dots) and ALDH^−^ (P4, purple dots) subpopulations were separated from SUM159 breast cancer cells by Flow Cytometry. For each sample, an aliquot of cells was stained under identical conditions with 15 mmol/L DEAB (a specific ALDH inhibitor) as an ALDH^−^ control.
(JPG)

Figure S4 **LLL12 (A), Stattic (B) and STAT3 ShRNA (C) also inhibited the cell viability of ALDH^−^ subpopulation.**
(JPG)

Figure S5 **Representative flow cytometry analysis of ALDH enzymatic activity and CD44/CD24 in SUM159 breast cancer cells was shown.** The percentage of ALDH+ cells is 4.4%, in which 93.7% are overlapped with CD44+/CD24− cells; the percentage of ALDH^−^ cells is 95.6%, in which 6.3% are overlapped with CD44+/CD24− cells.
(JPG)

Table S1 **Primer sequences and source information of STAT3 downstream target genes.**
(JPG)

Table S2 **The histological subtypes and other information about the tissue arrays.**
(JPG)

Table S3 **The effect of LLL12 on human protein and lipid kinases.**
(JPG)

Table S4 **The inhibition of LLL12 on STAT3 target genes expression in ALDH+ stem cell-like breast cancer cells was quantified and normalized to GAPDH.**
(JPG)
