# Supplementary figures and images for: Evaluation of STAT3 Signaling in ALDH+ and ALDH+/CD44+/CD24− Subpopulations of Breast Cancer Cells
Source: PLoS One. 2013 Dec 23;8(12):e82821. doi: 10.1371/journal.pone.0082821 (PMC3871589; doi:10.1371/journal.pone.0082821)

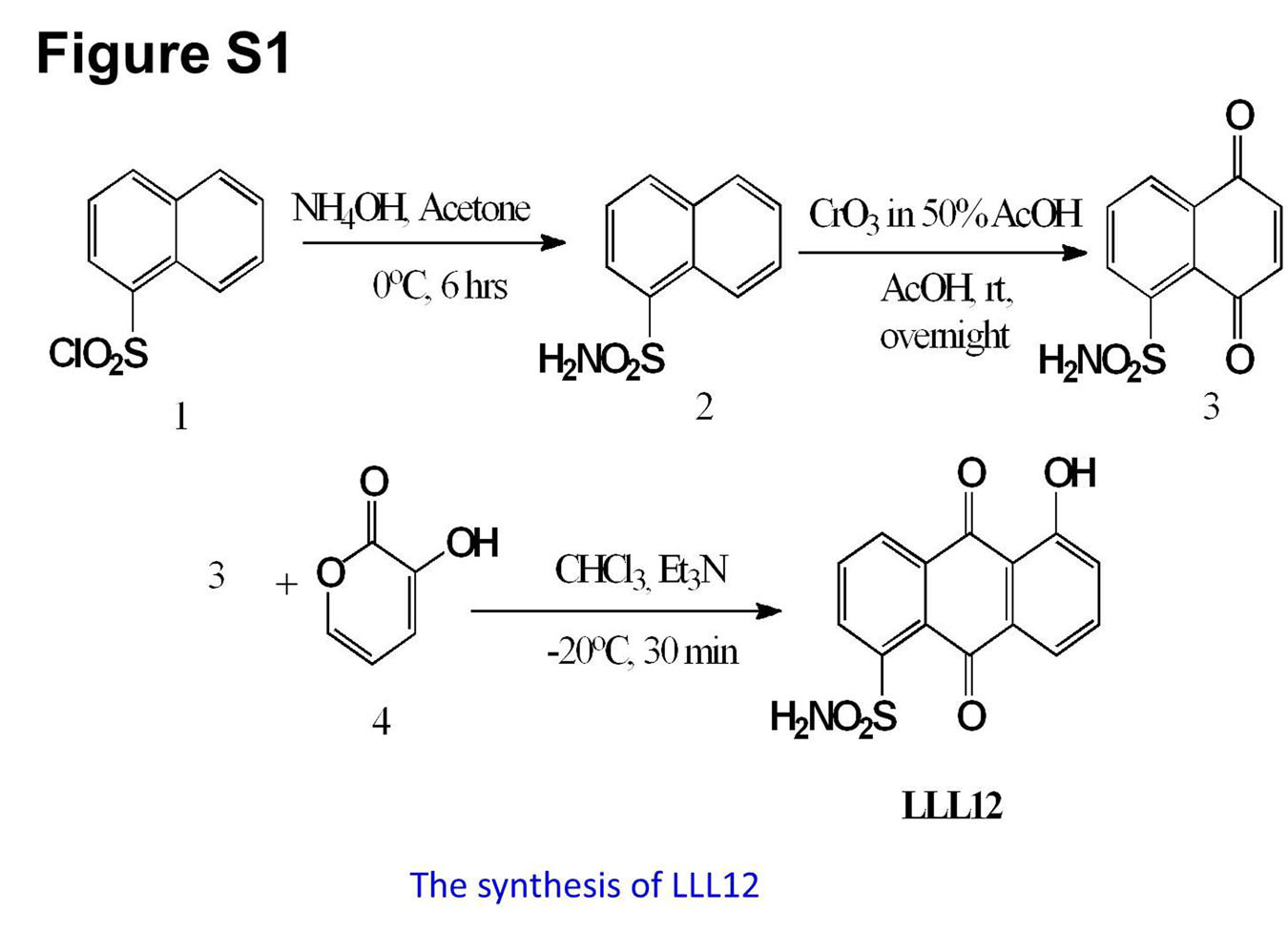

Supplement: Figure S1 — The synthesis of LLL12. (JPG) [file pone.0082821.s001.jpg]

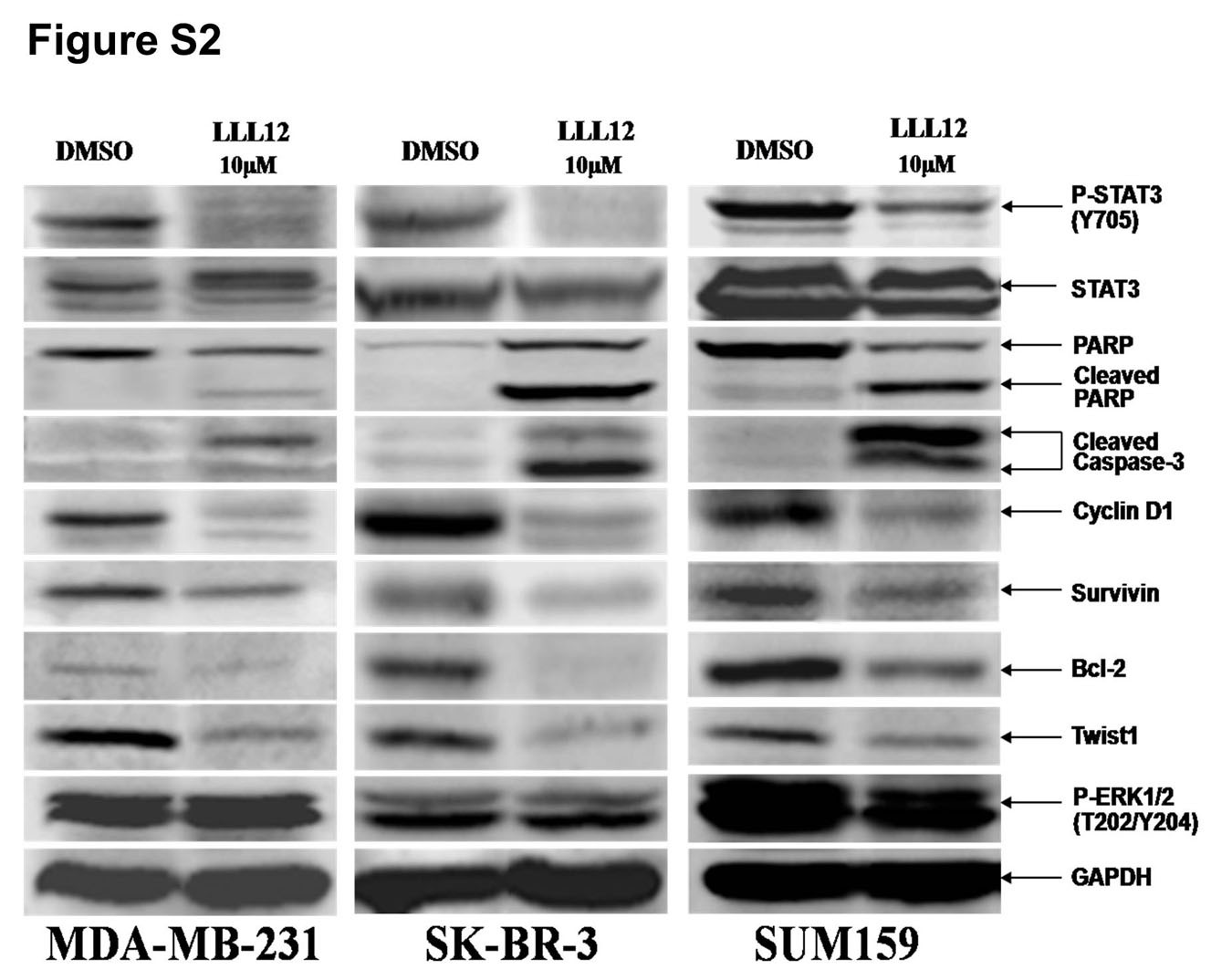

Supplement: Figure S2 — LLL12 inhibited STAT3 phosphorylation, and down-regulated STAT3-regulated genes, Cyclin D1, Survivin, Bcl-2 and Twist1, as well as induced apoptosis in un-seperated MDA-MB-231, SK-BR-3, and SUM159 breast cancer cells. LLL12 was synthesized in Dr. Pui-Kai Li’s laboratory (College of Pharmacy, The Ohio State University). Un-separated cells were treated with 10 µM of LLL12 or DMSO for 24 hours, and the phosphorylation of STAT3 (Y705), and ERK 1/2 (T202/Y204), and expression of STAT3 downstream genes, cleaved caspase-3, and PARP were detected by Western blots. (JPG) [file pone.0082821.s002.jpg]

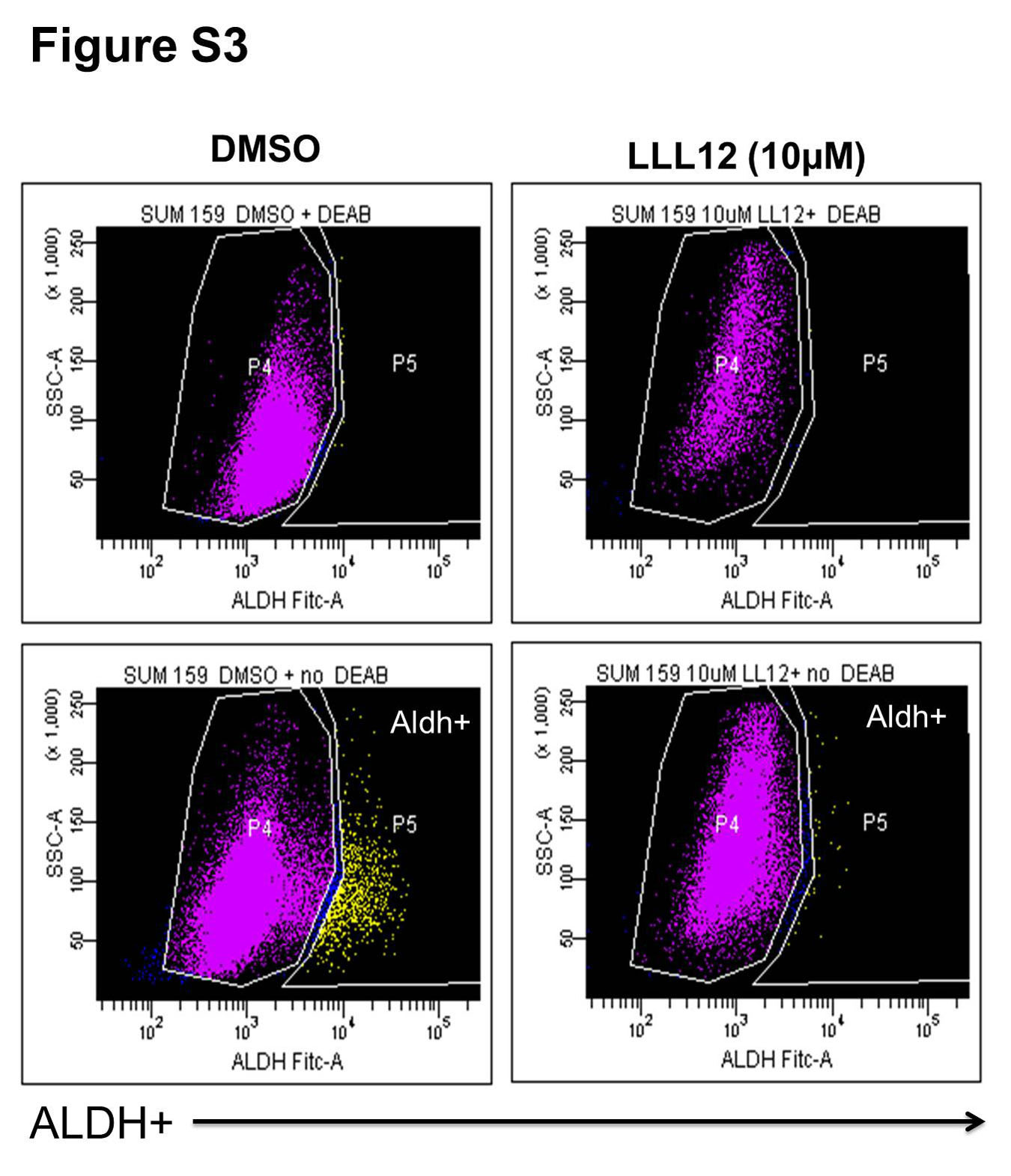

Supplement: Figure S3 — LLL12 (10 µM) decreased the percentage of ALDH+ subpopulation in SUM159 breast cancer cells. A representative example of flow cytometry analysis of ALDH+ cells in SUM159 breast cancer cells treated with LLL12. ALDH+ (P5, yellow dots) and ALDH− (P4, purple dots) subpopulations were separated from SUM159 breast cancer cells by Flow Cytometry. For each sample, an aliquot of cells was stained under identical conditions with 15 mmol/L DEAB (a specific ALDH inhibitor) as an ALDH− control. (JPG) [file pone.0082821.s003.jpg]

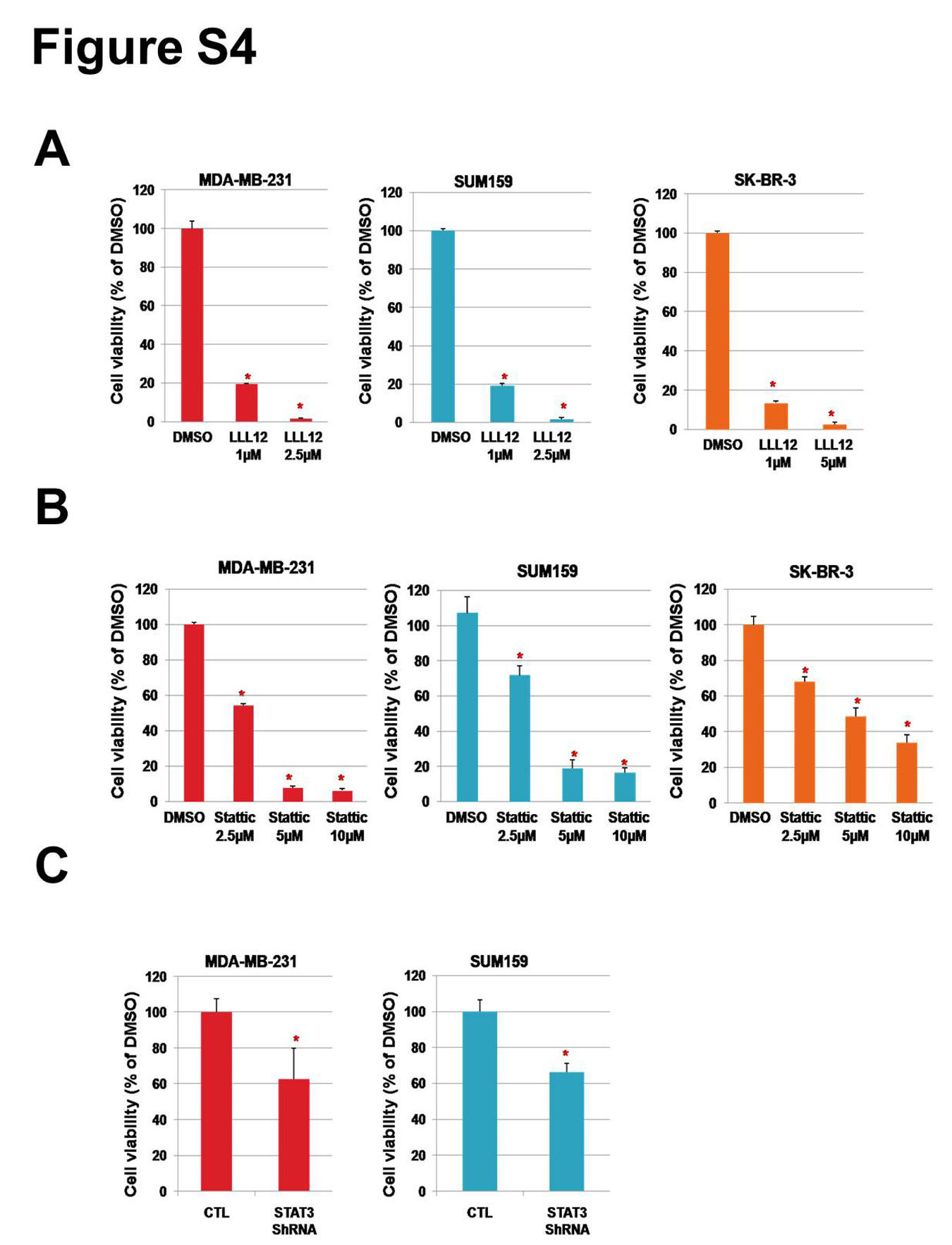

Supplement: Figure S4 — LLL12 (A), Stattic (B) and STAT3 ShRNA (C) also inhibited the cell viability of ALDH− subpopulation. (JPG) [file pone.0082821.s004.jpg]

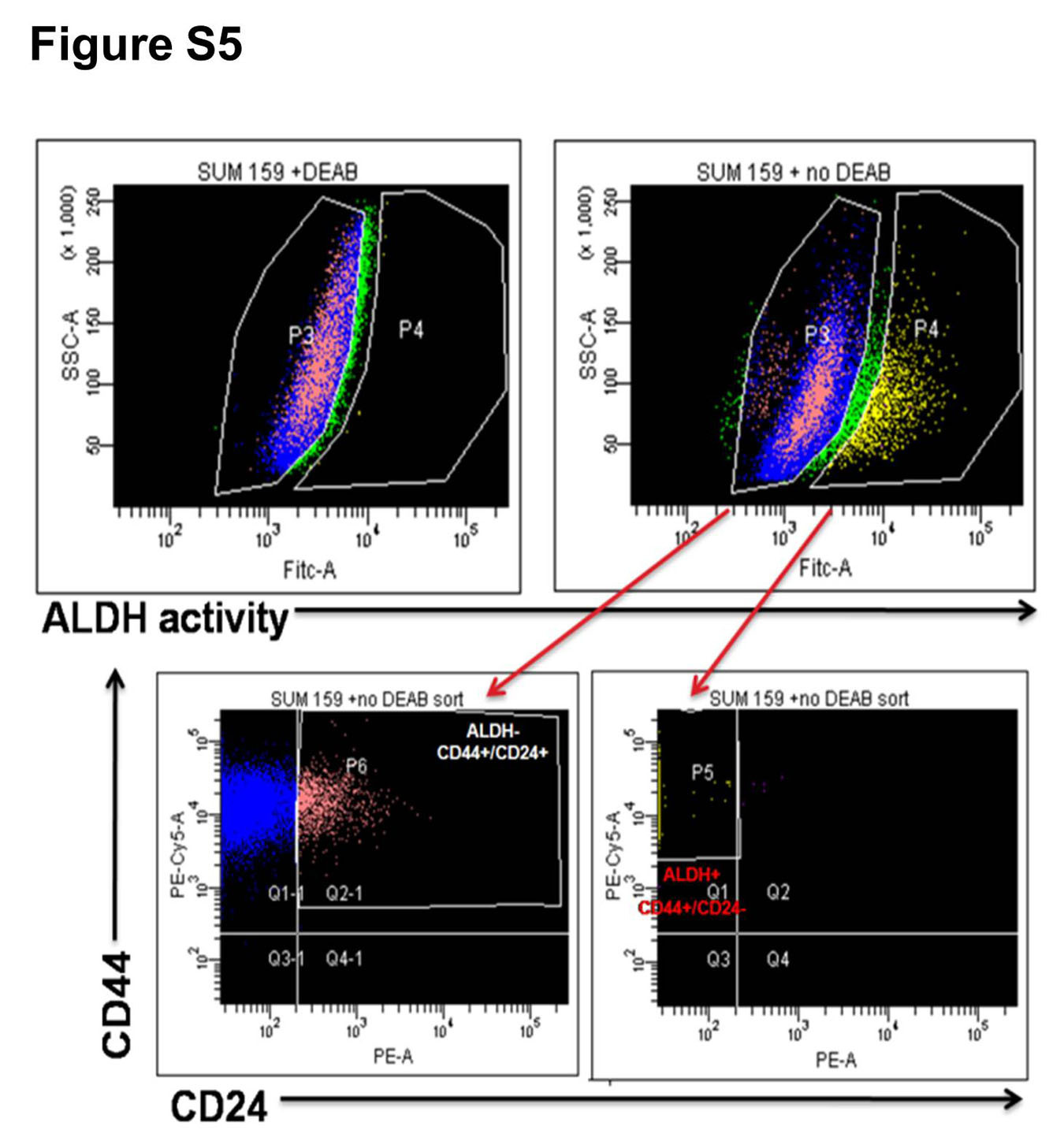

Supplement: Figure S5 — Representative flow cytometry analysis of ALDH enzymatic activity and CD44/CD24 in SUM159 breast cancer cells was shown. The percentage of ALDH+ cells is 4.4%, in which 93.7% are overlapped with CD44+/CD24− cells; the percentage of ALDH− cells is 95.6%, in which 6.3% are overlapped with CD44+/CD24− cells. (JPG) [file pone.0082821.s005.jpg]

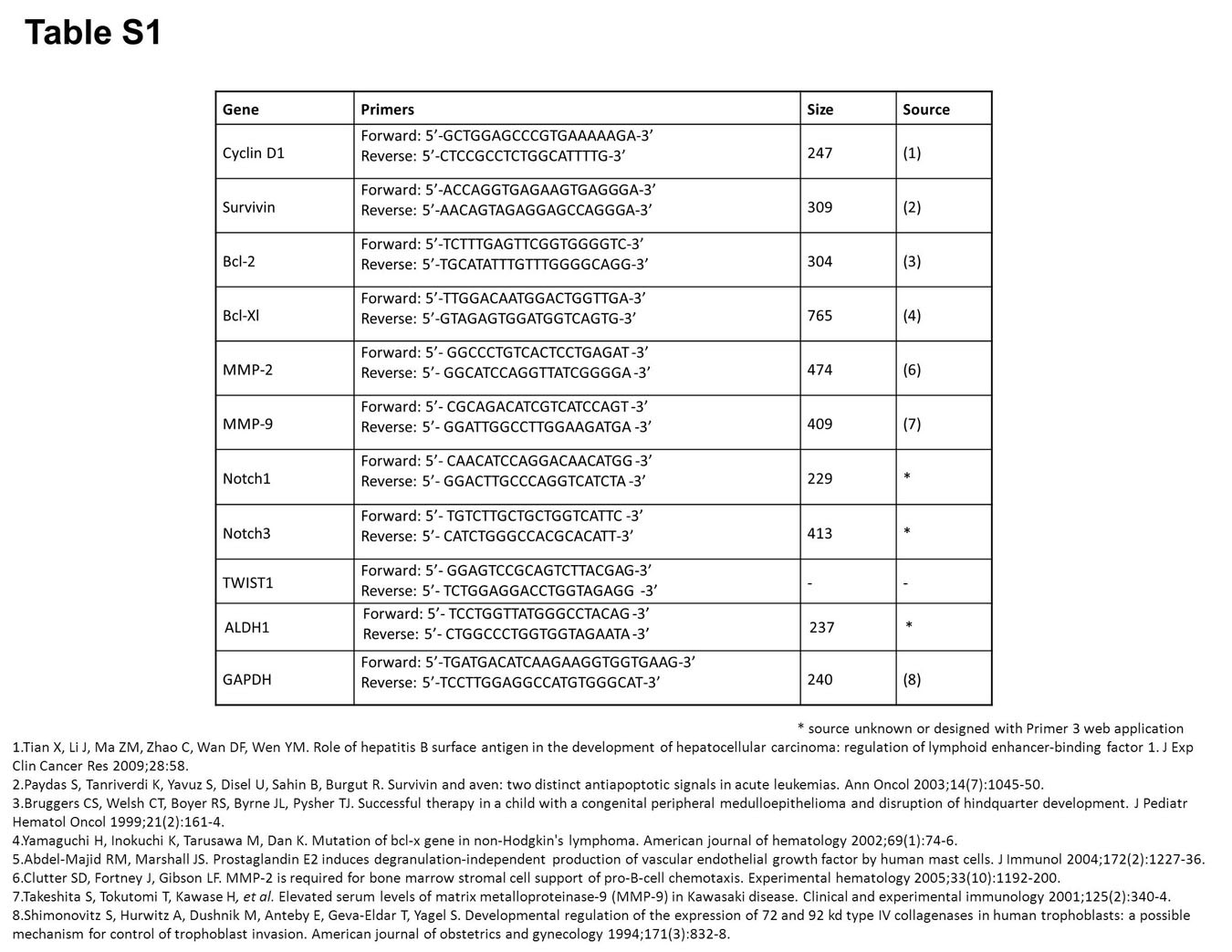

Supplement: Table S1 — Primer sequences and source information of STAT3 downstream target genes. (JPG) [file pone.0082821.s006.jpg]

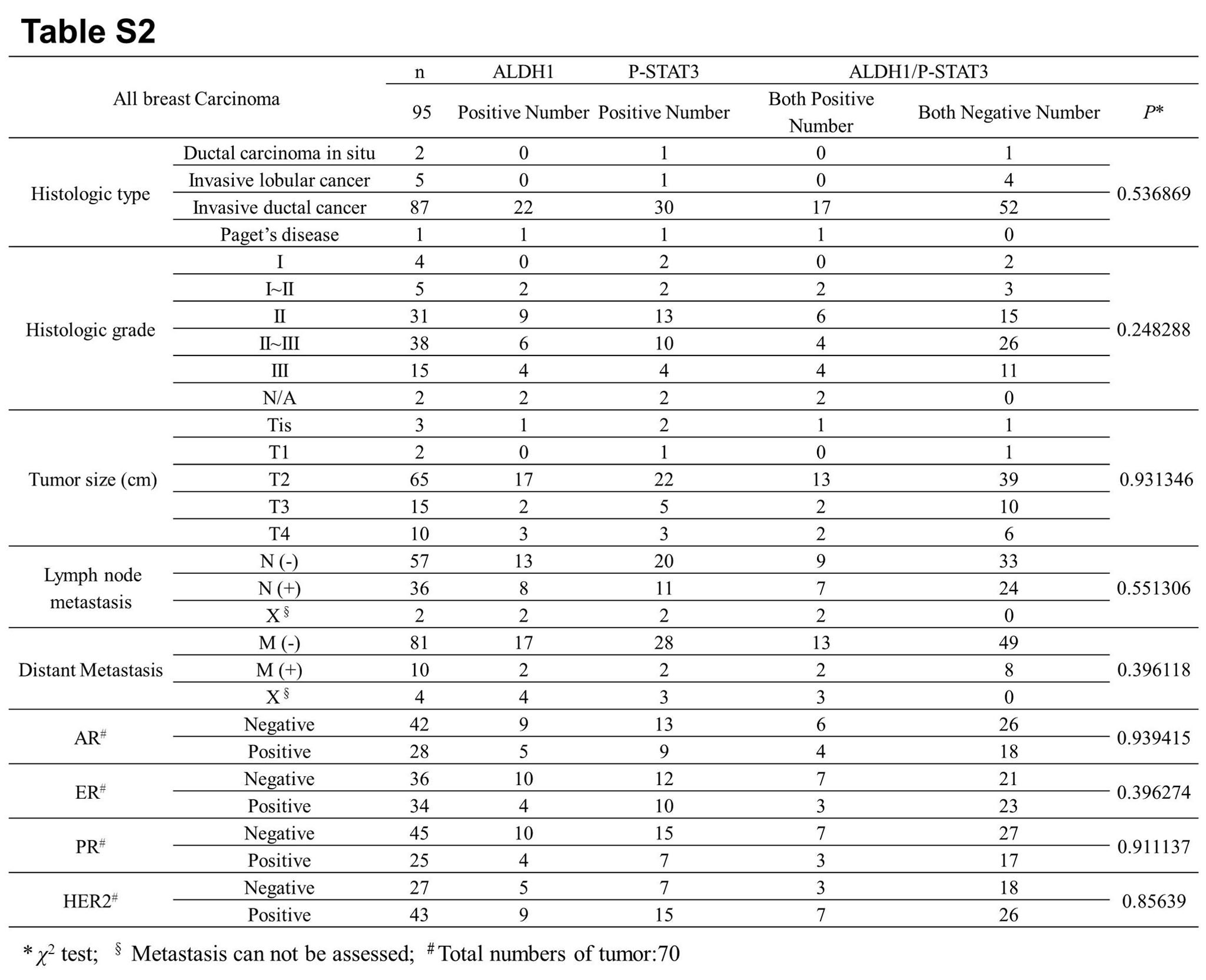

Supplement: Table S2 — The histological subtypes and other information about the tissue arrays. (JPG) [file pone.0082821.s007.jpg]

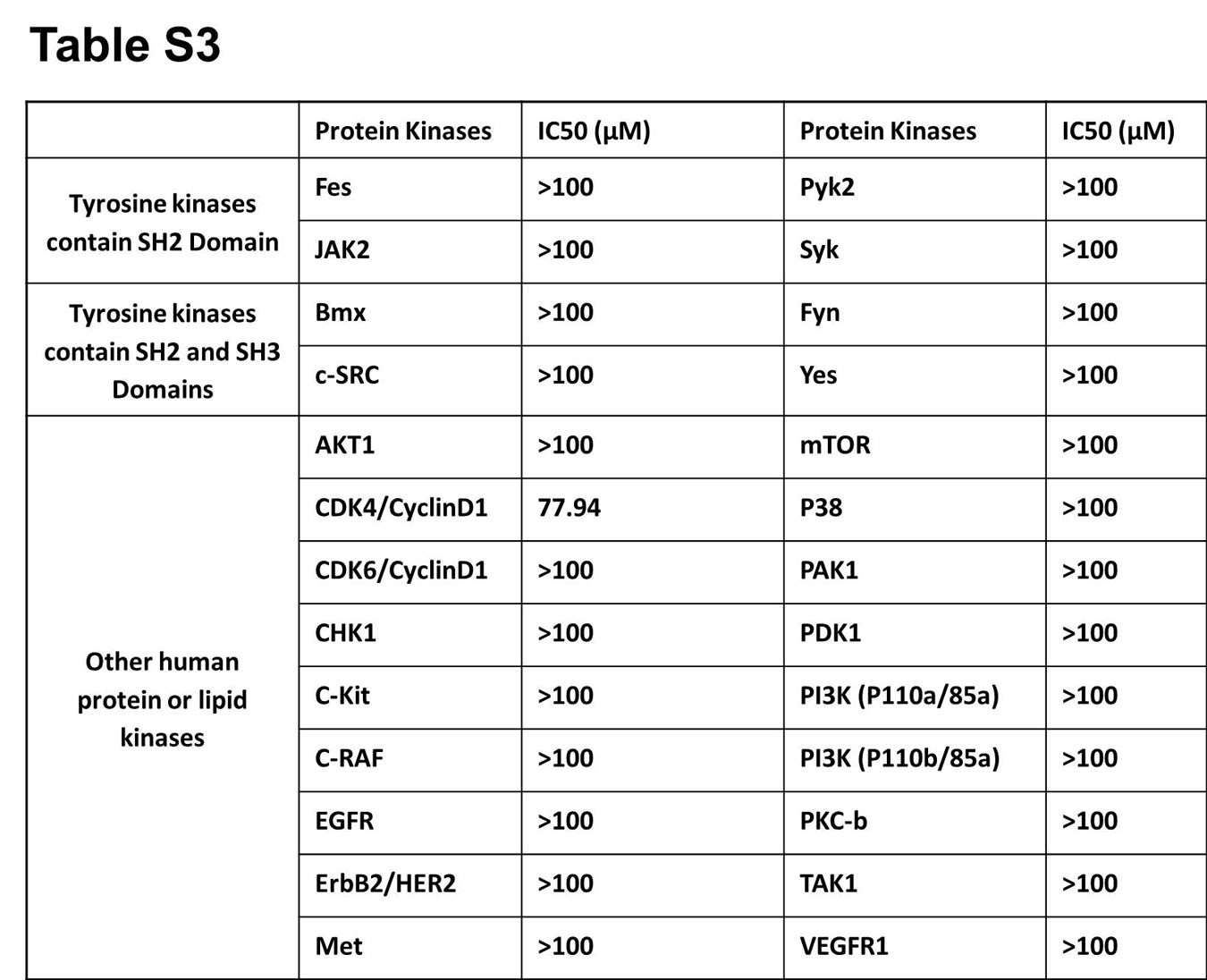

Supplement: Table S3 — The effect of LLL12 on human protein and lipid kinases. (JPG) [file pone.0082821.s008.jpg]

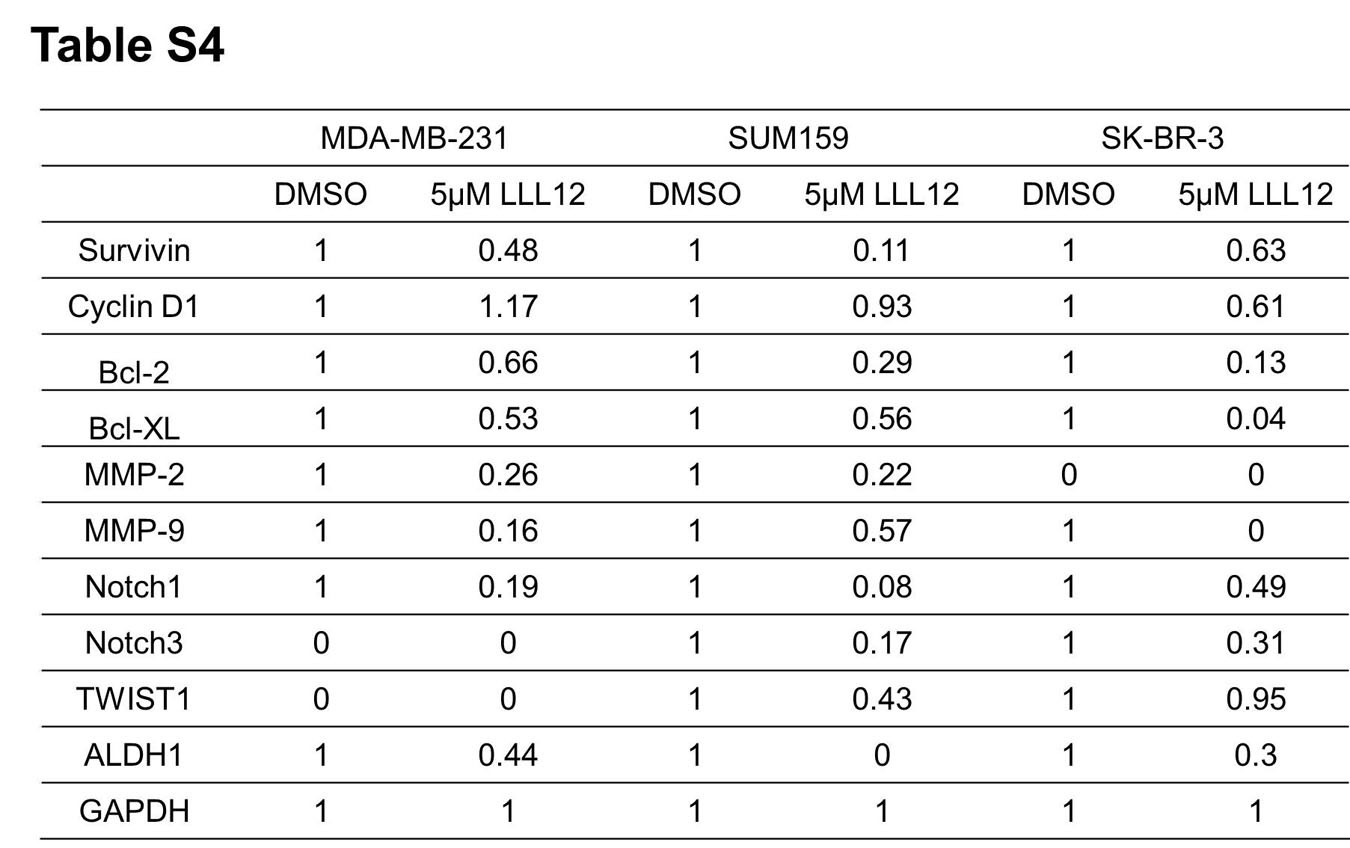

Supplement: Table S4 — The inhibition of LLL12 on STAT3 target genes expression in ALDH+ stem cell-like breast cancer cells was quantified and normalized to GAPDH. (JPG) [file pone.0082821.s009.jpg]
